# Approaching Reactive Arthritis Associated With Poor Prognostic Factors: A Case Report and Literature Review

**DOI:** 10.7759/cureus.13555

**Published:** 2021-02-25

**Authors:** Swetha Ann Alexander, Eunjung Kim, Ranadeep Mandhadi

**Affiliations:** 1 Internal Medicine, University of Connecticut, Farmington, USA; 2 Rheumatology, University of Connecticut, Farmington, USA

**Keywords:** reactive arthritis, nsaid, dmard, biologic therapies, poor prognostic markers, glucocorticoids, adalimumab, keratoderma blennorrhagicum, dactylitis

## Abstract

The aim of this paper is to review and discuss the background, common manifestations, differential diagnosis, and current treatment practices of reactive arthritis. The focus will be on the choice of therapy in patients with poor prognostic factors. A PubMed search was performed in March 2020 on reactive arthritis and revealed 137 articles. Fourteen case reports and four large-scale studies that are pertinent for discussion in terms of treatment of reactive arthritis over the past five years are reported along with poor prognostic markers. The first choice of therapy regardless of the number of poor prognostic markers is non-steroidal anti-inflammatory drugs (NSAIDs). The second choice of therapy appeared to be glucocorticoids in the oral as well as intra-articular forms. No correlation was detected between the need for systemic steroids and the number of poor prognostic factors present. The third choice of therapy appears to be disease-modifying anti-rheumatic drugs (DMARDs) (such as sulfasalazine) and their increasing use can be demonstrated over time. Novel therapies such as adalimumab have also been shown to be used and this shows a strong correlation with an increased number of poor prognostic factors. Reporting of these case reports and review of literature contribute to knowing more about reactive arthritis and help keep us up to date with newer therapies available when patients do not respond to conventional therapy. It was notable that the increased number of poor prognostic factors and non-responders have shown increased use of tumor necrosis factor inhibitors (TNFI) such as adalimumab.

## Introduction

Reactive arthritis (ReA) is a disease of a rare incidence and therefore internists and rheumatologists alike have limited encounters with diagnosing and treating the disease. We present a diagnostically and therapeutically challenging case of ReA. The patient had presented with multiple poor prognostic factors that posed unique challenges in treating this rare and intriguing disease. A literature review was performed to identify the best treatment modalities known to work in people with different prognostic factors. Given the rarity of ReA, we aim to review and discuss the background, common manifestations, differential diagnosis, and treatment of the disorder. 

## Case presentation

A 36-year-old African American man with a past medical history of herpes simplex virus (HSV) infection presented to the emergency department with a chief complaint of right knee pain and swelling of a couple of weeks. The symptoms began shortly after the patient returned from his trip to Minnesota where he was diagnosed with gonococcal urethritis by urine polymerase chain reaction (PCR) at a local emergency department. He had initially presented with a thick white urethral discharge and had been treated with one dose of cefixime 400 mg and doxycycline 100 mg twice a day for seven days. The patient returned to Connecticut and presumably finished his antibiotics treatment.

Over the next two weeks, the patient developed right knee pain and swelling and sought care in an outside emergency room. He was noted to have bilateral conjunctival injection in addition to right knee synovitis. He was given ibuprofen as well as erythromycin ophthalmic ointment. The patient returned the following day to the same emergency room due to minimal relief of his knee pain and this time was given prednisone taper for five days starting with 50 mg daily. Six days later, he presented to our emergency department with ongoing right knee pain.

Upon presentation, the patient also endorsed complaints of right ankle pain and swelling as well as left lateral hip pain. He had also complained of open sores on his penis, which per patient felt very similar to his prior HSV outbreaks. On further questioning, he noted feeling febrile but denied fatigue, weight loss, eye pain or redness, vision changes, rhinitis, ear discharge, chest pain, shortness of breath, abdominal discomfort, nausea, vomiting, diarrhea, dysuria or urethral discharge. Patient admitted to smoking about two to three cigarettes a day and drinking alcohol occasionally but denied use of illicit drugs. He has had multiple sexual partners over the past six months and did not use protection.

**Figure 1:**
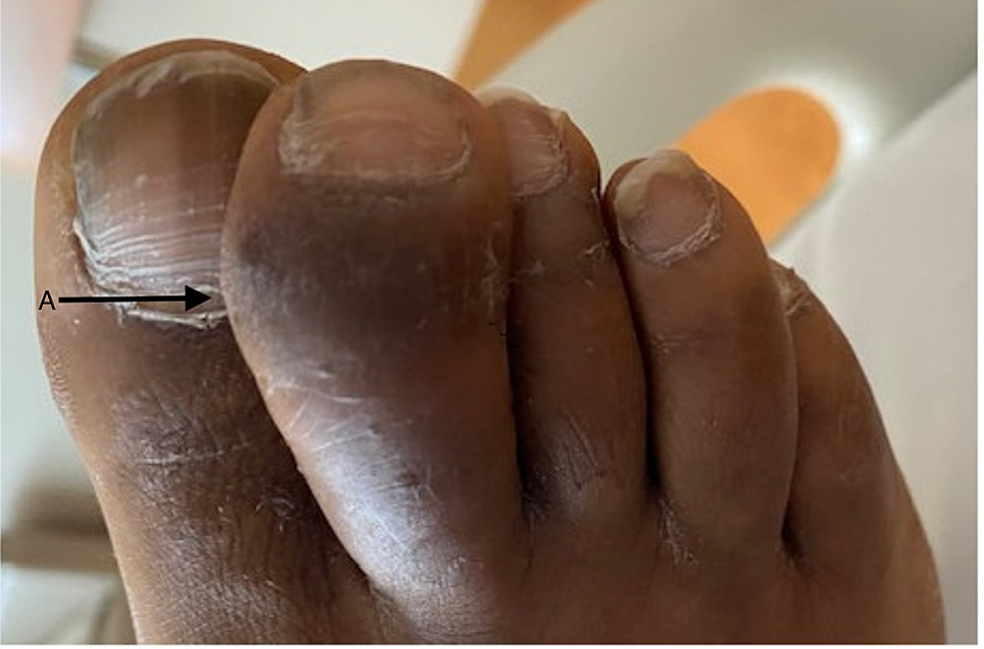
Dactylitis of the second toe As shown by black arrow (A)

**Figure 2:**
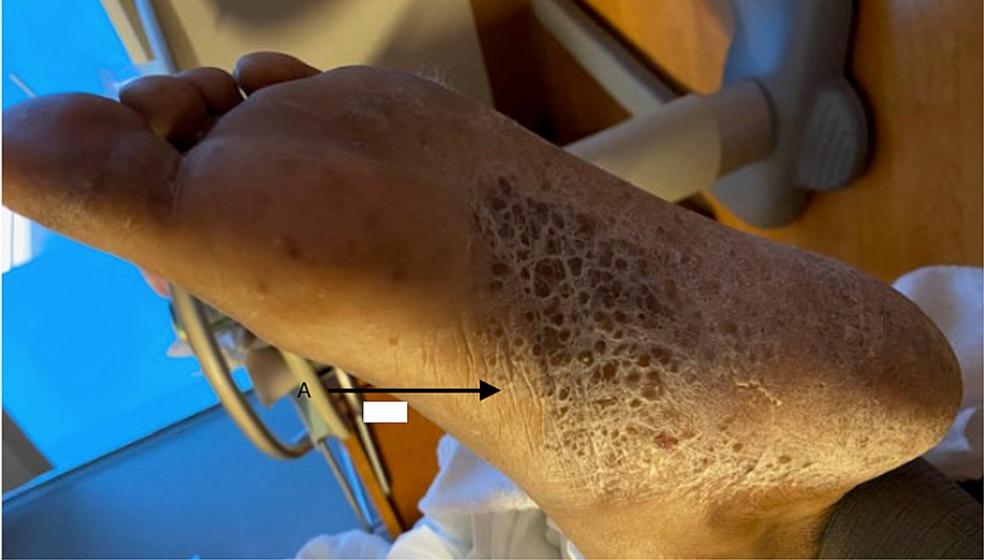
Keratoderma Blennorrhagicum As shown by black arrow (A)

On physical examination, he was febrile (102.9°F) and tachycardic (129 beats per minute). He was found to have bilateral conjunctival erythema with normal pupil size and light reactivity. Two non-tender, sub-centimeter, and elliptical-shaped dry ulcers with erythematous base were found on the hard palate of his mouth. There were a few mildly tender, non-purulent and sub-centimeter ulcers on his penis. There was no inguinal lymphadenopathy. His right knee was extremely warm, tender, and swollen. The range of motion was also limited due to pain. He had similar warmth, tenderness and swelling over the right ankle, and tenderness was noted over the left trochanteric bursa. Sausage-shaped swelling, consistent with dactylitis, of his right second toe was noted (Figure 1). Bilateral plantar surfaces were covered with hyperkeratotic hyperpigmented plaques consistent with keratoderma blennorrhagicum (Figure 2).

Lab work showed leukocytosis (white cell count of 24.0/µL) and significantly elevated inflammatory markers (erythrocyte sedimentation rate [ESR] of >130 mm/hr; C-reactive protein [CRP] of 262 mg/L). Right knee arthrocentesis revealed a turbid aspirate with 23,778 white blood cell count with 88% neutrophils. Gram stain was negative.

The patient was empirically started on ceftriaxone for disseminated gonococcal infection and azithromycin to cover for chlamydia. He was also given valacyclovir for herpetic ulcers on his penis. Despite treatment with antibiotics, he had persistent fevers and synovitis of the right knee and ankle. Repeat gonorrhea and chlamydia PCR returned negative. Human immunodeficiency virus (HIV), hepatitis B virus (HBV), hepatitis C virus (HCV), tuberculosis, syphilis, Lyme, Anaplasma, and Babesia also returned negative. Synovial cultures from initial as well as repeat arthrocentesis remained negative. Magnetic resonance imaging (MRI) of the right knee and ankle showed joint effusion but no other underlying abnormality was found. Human leukocyte antigen B27 (HLA-B27) was positive.

Given patient’s lower extremity predominant oligoarthritis, conjunctivitis, recent history of gonococcal urethritis, dactylitis, a classic rash of keratoderma blennorrhagicum and HLA-B27 positivity, a diagnosis of reactive arthritis was made. He was initially treated with ketorolac without much improvement in his joint symptoms. He was subsequently placed on two alternative non-steroidal anti-inflammatory drugs (NSAIDs) one after the other (indomethacin and diclofenac). A complete resolution of left trochanteric bursa pain was achieved with the NSAIDs, however debilitating pain in the right knee and ankle persisted. Finally, oral prednisone 40 mg daily was initiated with resolution of his fever and improvement of right knee and ankle synovitis. He was discharged home on prednisone with a plan for rheumatology outpatient follow-up. His total hospital stay was 12 days.

Patient was re-admitted to the hospital 10 days later due to persistent synovitis involving the right knee and ankle and a new-onset left knee synovitis. On further probing patient stated that he was non-adherent with taking prednisone at home. Repeat arthrocentesis again revealed aseptic inflammatory aspirate. He was started on intravenous methylprednisolone 40 mg twice a day along with sulfasalazine 500 mg twice a day. The patient's joint symptoms improved markedly over the next two days and he was discharged on oral prednisone. Unfortunately, he was lost to follow up upon discharge.

## Discussion

Multiple attempts to establish a guideline in order to define ReA after collaborating clinical and lab data have been made, however, none have been universally accepted or been validated. Current preliminary classification criteria for ReA, which has been widely used, is composed of two major and two minor criteria. A diagnosis of ‘definitive ReA’ is made if both major criteria and one relevant minor criterion are met. A diagnosis of ‘probable ReA’ is made when both major criteria without relevant minor criteria or one major criterion with one or more minor criteria are met [1]. The major and minor criteria of ReA are demonstrated in Table 1.

**Table 1:** Reactive Arthritis Diagnostic Criteria NAAT- Nucleic acid amplification test; PCR- Polymerase chain reaction

Major Criteria	Minor Criteria
Arthritis, with two of the following three findings: Asymmetric Monoarthritis or oligoarthritis Predominantly affecting lower limbs Preceding symptomatic infection 3 days to 6 weeks before the onset of arthritis: Enteritis (defined as diarrhea for at least 1 day) or urethritis (dysuria or discharge for at least 1 day)	Evidence of triggering infection: NAAT test from morning urine or urethral swab for Chlamydia Positive stool culture for enteric pathogens associated with reactive arthritis Evidence of persistent synovial infection: Positive immunohistology or PCR assay for Chlamydia

Clinical presentation of ReA varies widely ranging from the absence of symptoms to multi-systemic involvement. Usually, the course of disease starts with an infectious etiology leading to the development of arthritis (95%) after about one to four weeks [2]. Summarized in Table 2 are the potential clinical manifestations of ReA. Other causes of acute arthritis must be ruled out prior to the diagnosis of ReA (Table 3).

**Table 2:** Clinical Features of Reactive Arthritis Represented by Organ System

Organ Involvement	Typical Clinical Presentations
Musculoskeletal	Asymmetric lower limb oligoarthritis Dactylitis Sacroiliitis Enthesitis
Skin and Nail	Circinate balanitis Keratoderma Blennorrhagica Psoriatic onychodystrophy Painless ulcers in the mouth
Eye	Conjunctivitis Corneal ulceration Episcleritis Keratitis Uveitis
Genitourinary	Cervicitis/Salpingitis/Vulvovaginitis in women Urethritis and Prostatitis in men
Gastrointestinal	Acute diarrhea resembling inflammatory bowel disease
Heart (Rare)	Ascending aortitis Conduction abnormalities

**Table 3:** Differential Diagnosis for Reactive Arthritis WBC- white blood cell; RF- rheumatoid Factor; CCP- cyclic citrullinated peptide; ESR- erythrocyte sedimentation rate; CRP- C-reactive protein; STI- sexually transmitted infection; SLE- systemic lupus erythematosus

Diagnosis	Characteristic Features
Septic Arthritis	Mono or oligoarticular involvement with joint effusion showing elevated WBC counts (e.g. > 50,000 cells/microL) and positive synovial fluid cultures for causative organism.
Gout	Monosodium urate crystals present on synovial fluid analysis with elevated WBC counts
Pseudogout	Calcium pyrophosphate crystals present on synovial fluid analysis with elevated WBC counts
Rheumatoid Arthritis Flare	Symmetric polyarticular involvement with positive serology (RF/anti-CCP antibodies), elevated acute phase reactants (ESR/CRP)
Psoriatic Arthritis	Oligo- or polyarthritis with onycholysis and psoriatic skin lesion
Lyme Arthritis	Mono- or oligoarthritis with positive Lyme serology
STI related arthritis	Based on the serology of the virus
Lupus Arthritis	Migratory, polyarticular and symmetric involvement with systemic features of SLE
Inflammatory bowel disease-associated arthritis	Peripheral arthritis with spondylitis and history of inflammatory bowel disease
Sarcoid arthropathy	Oligo or polyarthritis associated with hilar adenopathy and erythema nodosum

Clinical course of reactive arthritis is variable. It can spontaneously resolve or progress to chronic arthritis which is defined as persistence of symptoms for greater than six months. More than half of the cases that have a chronic course are usually triggered by genitourinary organisms as compared to enteric organisms. This can be secondary to increased rates of reinfection with genitourinary organisms such as *Chlamydia trachomatis* and *Neisseria gonorrhea*.

Certain factors on presentation are indicative of poor prognosis as they point towards a more severe spectrum of the disease. These factors include male sex, age of disease onset less than 16 years, HLA-B27 positivity, heel and foot involvement, arthritis of the hip, lumbar spine stiffness, dactylitis, oligoarthritis, ESR greater than 30 mm in the first hour, and poor response to NSAIDs. 

A multifaceted approach to treatment is required based on the natural course of the disease and the clinical course of the patient. The goals of treatment are threefold: 1) reduce pain and inflammation, 2) minimize disability, and 3) prevent relapse or progression to chronic disease. Initially, the treatment of an underlying genitourinary infection with antibiotics is essential but the same approach does not apply for enteric infections with rare exceptions (e.g. *Clostridium difficile*). 

Considering the long hospital course of our patient and his multiple poor prognostic factors, we sought out to review what the recent treatment practice patterns of ReA were. We especially wanted to examine the impact of poor prognostic factors on physicians’ treatment regimens and the need for escalation of therapy to disease-modifying anti-rheumatic drugs (DMARDs) or biologics in such cases. Additionally, we hoped to investigate if there was any correlation between early initiation of prednisone (prior to use of NSAIDs) and decreased requirement of DMARDs or biologics use.

A review of the literature was performed through a Medical Subject Headings (MeSH) search of PubMed for all articles on ‘Reactive Arthritis’ published within the last five years. It revealed 137 articles as of March 2020 from which case reports and large-scale data reported in English were included. We analyzed 14 case reports and four large-scale studies that are pertinent for our discussion. The case reports and the large studies are displayed in Table 4 [3-16] and Table 5 [17-20], respectively. We risk-stratified the case reports by poor prognostic factors and analyzed the treatment options in the order they were used in each case study.

**Table 4:** Case Reports of Reactive Arthritis with Choices of Treatment and Poor Prognostic factors ReA- reactive arthritis; HLA-B27- human leucocyte antigen- B27; ESR- erythrocyte sedimentation rate; NSAIDs- nonsteroidal anti-inflammatory agents; BCG- bacillus Calmette–Guerin; LGV- lymphoma granuloma venerum

Serial Number	Author	Year	Country Of Study	Diagnosis	Choices of therapy in the order of use	Poor prognostic factors
1	Sumiyoshi et al. [15]	2019	Japan	ReA and gout	Loxoprofen, Salazosulfapyridine, Adalimumab	Male sex, Foot involvement, Elevated ESR, HLA-B 27 positivity, Poor response to NSAIDs
2	Hoversten et al. [9]	2018	USA	ReA	NSAID, Prednisone, Sulfasalazine, Adalimumab	Male sex, Oligoarthritis, Elevated ESR, HLA-B27 positivity, Poor response to NSAIDs
3	Courcoul et al. [4]	2017	France	ReA secondary to Salmonella enteritidis	Ketoprofen, Sulfasalazine, Etanercept	Oligoarthritis, Dactylitis, Elevated ESR, HLA-B27 positivity, Poor response to NSAIDs
4	Michiels et al. [12]	2019	Belgium	ReA	Local glucocorticoid, Etoricoxib, Sulfasalazine	Male Sex, Oligoarthritis, Dactylitis, HLA-B27 positivity
5	Erre et al. [6]	2019	Italy	Hafnia alvei induced ReA	NSAID, Ciprofloxacin	Male sex, Oligoarthritis, Elevated ESR, Poor response to NSAIDs
6	Hsing et al. [10]	2017	Australia	ReA	Naproxen, Prednisolone, Sulfasalazine	Male sex, Elevated ESR, HLA-B 27 positivity, Poor response to NSAIDs
7	Nishizaki et al. [14]	2016	Japan	ReA	NSAID, Prednisolone taper, Methotrexate	Male sex, Oligoarthritis, HLA-B 27 positivity, Poor response to NSAIDs
8	Kawahara et al. [11]	2018	Japan	ReA secondary to Neisseria meningitidis	Antibiotics, Prednisolone, Methotrexate	Male sex, Oligoarthritis, HLA-B27 positivity
9	Eguchi et al. [5]	2019	Japan	Haemophilus parainfluenzae induced ReA	Ampicillin	Oligoarthritis, Elevated ESR
10	Yoshimura et al. [16]	2018	Japan	Intravesical bacillus Calmette–Guerin (BCG) induced ReA	NSAID therapy, Methotrexate, Salazosulfapyridine, Prednisolone	Male sex, Oligoarthritis
11	Coelho et al. [3]	2017	Portugal	ReA	Antibiotics, NSAID, Sulfasalazine	Oligoarthritis, HLA-B27 positivity
12	Ng et al. [13]	2015	Italy	ReA after intravesical BCG instillations	NSAID	Male sex, Oligoarthritis
13	Gałąska et al. [8]	2019	Poland	Yersinia induced ReA and Aortitis	Naproxen, Oral corticosteroids, Cyclophosphamide	Oligoarthritis
14	Foschi et al. [7]	2016	Italy	ReA secondary to LGV infection	Doxycycline, Ketoprofen	Dactylitis

**Table 5:** Large Scale Studies Reporting Treatment Patterns of Reactive Arthritis NSAIDs- nonsteroidal anti-inflammatory drugs; DMARDS- disease-modifying antirheumatic drugs; TNF- tumor necrosis factor; HLA-B27- human leucocyte antigen- B27; ReA- reactive arthritis

Serial Number	Author	Year of Publication	Country of Study	Type of Study	Survey Results
1	Hayes et al. [19]	2019	Canada	Cross-sectional Survey	Interview of rheumatologists revealed the following: 97% supported the use of NSAIDs as initial choice; 65% supported the use of intraarticular corticosteroid injections after failure with NSAIDs; 45% used DMARDS; 66% used TNF alpha inhibitors as a last resort
2	Ferrer et al. [18]	2019	Guatemala	Prospective Cohort Study	32 patients with ReA out of whom 19% were Males and 6% had HLA B27 positivity followed up for 2 years for symptomatic improvement: 97% treated with NSAID; 6% required glucocorticoids (route unspecified). 47% of ReA cases were symptomatic at the end of 2 years
3	Courcoul et al. [17]	2018	France	Retrospective Study	Two cohorts of patients between 1986–1996 and 2002–2012 were studied. 31 and 27 ReA patients respectively were identified. Poor prognostic factors were: Male sex (71% vs 74%); Oligoarthritis (42% vs 44%); Sacroiliitis (10% vs 22%); HLA-B27 positivity (64% vs 57%; p-value of 0.57) respectively. NSAIDs usage: 87% vs 96%, respectively (p-0.36); Oral glucocorticoids usage: 26 % vs 67%, respectively (p<0.01); DMARD usage: 16% vs 63% respectively (p<0.01); Anti-TNF usage: 0% vs 5% respectively (p-0.02). Complete recovery: 42% (1986-1996 cohort) vs 26% (2002 -2012 cohort). Spondyloarthritis occurrence: 16% (1986-1996 cohort) vs 55% (2002-2012)
4	Brinster et al. [20]	2016	France	Retrospective Study	Patients with ReA were studied in two cohorts between January 1984 to December 1993, and from January 2004 to December 2013. Poor prognostic factors overall in both cohorts were: Male sex (83.9%); Dactylitis (19.4%); HLA-B27 positivity (64.3%). Antibiotic use: 77% (1984-1993) vs 93 % (2004 to 2013); 91.8% of the entire cohorts were given NSAIDs. DMARD use: 36% (1984-1993) vs 62% (2004-2013); Biologic agent use: 0% (1984-1993) vs 45% (2004-2013). No Symptomatic improvement difference between the groups

Among the 14 case reports mentioned above, 11 case reports documented the use of NSAIDs as the first choice of therapy. NSAID therapy was a second choice seen in case number 4 (Table 4), after non-relief with intra-articular glucocorticoid use [12]. This supports that NSAIDs appears to be the first choice of therapy.

Five case reports documented the use of oral steroids as the second choice of therapy after non-relief with NSAIDs [8-11,14,16]. Case number 8 (Table 4) initiated oral steroids as a first line therapy, followed by DMARD therapy for additional symptom control [11]. Case number 10 chose oral steroid after trial of NSAID and DMARD therapy for symptomatic management [16]. Out of these five cases which required oral steroids after failure with NSAIDs, one case presented with five poor prognostic factors, two cases with four poor prognostic factors, one with three poor prognostic factors and one with two poor prognostic factors [8-10,14]. They uniformly required the use of DMARDs. This depicts that higher number of prognostic factors lead to escalation of therapy from NSAID to glucocorticoid use.

Interestingly, case number 1 and case number 10 (Table 4) initiated DMARDs and TNF inhibitors (TNFI) even before the use of oral steroids [15,16]. Case numbers 1 and 2 (Table 4) have demonstrated the use of adalimumab [9,15]. It is notable to mention that five or greater prognostic factors were identified in both cases in which adalimumab was used. The use of TNFI is increasing over the years and symptomatic improvement with its use is seen in about 20% of cases [17,20].

Large-scale studies are reported in Table 5. A cross-sectional survey in Canada (study 1, Table 5) showed that intra-articular glucocorticoid treatment has been used widely among 65% of the rheumatologists [19]. A prospective cohort study from Guatemala (study 2, Table 5) showed that 6% of their cohort required glucocorticoids treatment after non-relief with NSAIDs therapy alone and symptomatic improvement was seen in 53% of the patients by the end of treatment with NSAID and glucocorticoid [18]. The route of glucocorticoids was not specified in this study. Table 5 includes two retrospective studies (studies 3 and 4) which compare the trend of treatment choices in the different time periods [17, 20]. A trend favoring the prevalent usage of DMARDs and biologics is seen after the year 2000.

Over the years, therapy for recalcitrant ReA has expanded which may reflect the general advancement of rheumatologic armamentaria. However, it appears NSAIDs are still used most commonly as first-line therapy in ReA and increased use of DMARDs or biologic does not always lead to resolution of symptoms. Initiation of early systemic steroid therapy does not seem to lessen the requirement of DMARDs when associated with poor prognostic factors. Even as little as two poor prognostic factors were associated with the use of DMARDs. Unfortunately, the timing of each treatment regimen in respect to symptom onset was not clearly denoted in the case reports and therefore, the effect of early treatment with systemic glucocorticoids on the need for subsequent escalation of treatment is unclear.

There are a few limitations in our literature search, as follows: First, only English language written articles were included so underreporting of ReA cases was inevitable. Secondly, no strict inclusion or exclusion criteria for the mentioned articles were in place. Articles that were included were those in which patients were diagnosed with ReA and treated for it. The aim was to review the treatment utilized and outcomes observed in ReA patients over the past five years. Third, they only include retrospective findings from the cases and large-scale studies and cannot fully assess for superiority or inferiority of each treatment regimen compared to each other and come with inherent limitations associated with retrospective studies.

The major strengths of this study are that it provides a single source to review many of the recently published case reports and large-scale studies focusing on the treatment choices and their relation to poor prognostic factors and symptomatic relief. It also discusses the treatment trends over time while reinforcing that NSAIDs are still the first line of therapy.

## Conclusions

ReA is a rare form of arthritis and awareness of this condition is crucial for accurate diagnosis and initiation of appropriate therapy in a timely manner. We presented a case of ReA with multiple poor prognostic factors and reviewed various aspects of ReA including clinical manifestations and appropriate management strategies. Given the rarity of ReA, there is a lack of randomized controlled studies on treatment options. NSAIDs still continue to be the first treatment choice. Poor prognostic factors usually lead to escalation of therapy including treatment with the biologics. Initiation of systemic steroids did not necessarily prevent the need to escalate therapy to DMARDs or biologics, especially when associated with poor prognostic factors. There is a need to establish universally acceptable guidelines for identifying ReA so that prospective studies can be done to ascertain the best treatment strategy.
